# Scavenging Capacity of Extracts of *Arrabidaea chica* Leaves from the Amazonia against ROS and RNS of Physiological and Food Relevance

**DOI:** 10.3390/antiox11101909

**Published:** 2022-09-27

**Authors:** Francilia Campos de Siqueira, Anna Paula Pereira Barbosa-Carvalho, Deusa do Socorro Teixeira Costa Leitão, Kalebe Ferreira Furtado, Gilson Celso Albuquerque Chagas-Junior, Alessandra Santos Lopes, Renan Campos Chisté

**Affiliations:** 1 Graduate Program of Food Science and Technology, Institute of Technology, Federal University of Pará (UFPA), Belém 66075-110, Brazil; 2 School of Biotechnology, Institute of Biological Sciences (ICB), Federal University of Pará (UFPA), Belém 66075-110, Brazil; 3 School of Food Engineering, Institute of Technology, Federal University of Pará (UFPA), Belém 66075-110, Brazil; 4 Renan Campos Chisté, Faculdade de Engenharia de Alimentos (FEA), Instituto de Tecnologia (ITEC), Federal University of Pará (UFPA), Rua Augusto Corrêa, 01-Guamá, Belém 66075-110, Brazil

**Keywords:** antioxidant capacity, Amazonian plant, reactive oxygen species, reactive nitrogen species, green solvents, phenolic compounds

## Abstract

*Arrabidaea chica*, a medicinal plant found in the Amazon rainforest, is a promising source of bioactive compounds which can be used to inhibit oxidative damage in both food and biological systems. In this study, the in vitro scavenging capacity of characterized extracts of *A. chica* leaves, obtained with green solvents of different polarities [water, ethanol, and ethanol/water (1:1, *v*/*v*)] through ultrasound-assisted extraction, was investigated against reactive oxygen (ROS) and nitrogen (RNS) species, namely superoxide anion radicals (O_2_^•^^−^), hydrogen peroxide (H_2_O_2_), hypochlorous acid (HOCl), and peroxynitrite anion (ONOO^−^). The extract obtained with ethanol–water presented about three times more phenolic compound contents (11.8 mg/g) than ethanol and water extracts (3.8 and 3.6 mg/g, respectively), with scutellarein being the major compound (6.76 mg/g). All extracts showed high scavenging efficiency against the tested ROS and RNS, in a concentration-dependent manner with low IC_50_ values, and the ethanol–water extract was the most effective one. In addition, all the extracts were five times more efficient against ROO^•^ than Trolox. Therefore, the extracts from *A. chica* leaves exhibited high promising antioxidant potential to be used against oxidative damage in food and physiological systems.

## 1. Introduction

In biological systems, reactive oxygen (ROS) and nitrogen (RNS) species play an essential role in maintaining the body’s normal physiological condition [1,2]. However, the overproduction of ROS/RNS, as a result of physiological disorders, combined with the deficiency of endogenous and exogenous antioxidants in the human body, induces oxidative and nitrosative stress, damaging basic components for function and cell survival, which may be related to the occurrence of several chronic degenerative diseases, such as cardiovascular diseases, diabetes, Alzheimer’s, and cancer [3,4,5].

In addition to the deleterious effect caused to physiological systems, oxidative reactions induced by the presence of ROS and RNS can also be observed in food systems. The oxidative effects of ROS can occur during food harvesting, storage, and processing, producing off-flavors as well as changes in color and texture, which adversely affect the overall quality and food safety due to the degradation of essential fatty acids, amino acids, and vitamins, and might promote the formation of toxic and carcinogenic compounds [6,7,8,9]. Due to these harmful effects, the inhibition of ROS and RNS in food systems is highly desirable and, therefore, the use of antioxidants as food additive (natural or artificial) can be seen as a very efficient strategy to delay oxidative reactions that decrease the shelf life of processed foods.

Scientific evidences suggest that antioxidants obtained from natural sources, such as fruits and other vegetables, are efficient replacements to synthetic antioxidants with the same purpose of preventing the formation of undesirable oxidation products, improving the quality of food products, consequently improving the life quality of consumers [10,11,12,13,14]. Scientific data, combined with the perception of modern consumers on the need for foods with natural and healthier ingredients in the formulation, resulted in a growing trend at the scientific level and in the food industry for the incorporation of natural additives.

In this context, the Amazon is known as one of the richest biomes in the world concerning biodiversity, including several claimed medicinal plants, which present a number of bioactive molecules in their composition, with promising biological activities [15]. Among these plants, *Arrabidaea chica* (Humb. & Bonpl.) B. Verlot (pariri), from the Bignoniaceae family, is potentially rich in phenolic compounds that has been used to treat various diseases [16,17,18]. Some studies have also reported anti-inflammatory, antimicrobial, and antifungal properties for the leaves of *A. chica* [15,19].

According to a recent study published by our research group, *A. chica* leaves were considered to be promising sources of phenolic compound contents, with feruloyl hexose, scutellarin, apigenin glucuronide, flavone-glucuronyl derivative, scutellarein, methyl apigenin glucuronide, and apigenin being tentatively identified, in addition to exhibiting high quenching abilities against singlet oxygen (^1^O_2_) [20], a highly reactive ROS derived from molecular oxygen.

Other studies also showed that the extract of *A. chica* leaves has higher antioxidant capacity when compared to scutellarein and apigenin isolated from the leaf, as evaluated by DPPH (2,2-diphenyl-1-picrylhydrazyl), β-carotene/linoleic acid, total reactive antioxidant potential (TRAP), and peroxyl radical (ROO^●^) assays [21,22]. Furthermore, in an in vitro cellular study, post-treatment with *A. chica* in L929 fibroblasts decreased oxidative damage by inhibiting intracellular ROS and mitochondrial superoxide anion radicals (O_2_^•−^) induced by UV-A and UV-B irradiation [5]. However, to the best of our knowledge, there are no reports in the literature regarding the direct antioxidant effect of *A.chica* extracts against other ROS and RNS commonly found in physiological and food systems, namely O_2_^•−^, hydrogen peroxide (H_2_O_2_), hypochlorous acid (HOCl), and peroxynitrite anion (ONOO^−^). Such information may stimulate relevant applicability to extracts obtained from *A. chica* leaves to confer antioxidant action in several applications of interest to food, pharmaceutical, and cosmetic industries.

On the other hand, it should be taken into account that several factors can significantly affect the phytochemical content and antioxidant capacity of natural extracts, including the type and polarity of the solvent used [23,24]. In addition, there is a greater appeal for the use of solvents that favor green chemistry, associated with the use of emergent extraction techniques, for example, ultrasound-assisted extraction (UAE), which is safer and less time-consuming, promotes high-efficiency extractions, and can minimize environmental impacts [25,26].

Therefore, our study investigated the in vitro antioxidant capacity of *A. chica* extracts, obtained through UAE technique using three green solvents of different polarities [water, ethanol and ethanol/water mixture (1:1, *v*/*v*)] against the ROS and RNS of food and physiological relevance, namely O_2_^•−^, H_2_O_2_, HOCl, ^1^O_2_, ROO^•^, and ONOO^−^ (in the presence and absence of NaHCO_3_ to simulate physiological conditions).

## 2. Materials and Methods

### 2.1. Chemicals

Scutellarin, apigenin, quercetin, L-ascorbic acid, quercetin 6-hydroxy-2,5,7,8-tetramethylchroman-2-carboxylic acid (trolox), lucigenin, fluorescein Dihydrorodamine (DHR), phenazine methosulfate (PMS), nitroblue tetrazolium chloride (NBT), α,α′-Azodiisobutyramidine dihydrochloride (AAPH), dimethyl sulfoxide (DMSO), sodium hypochlorite solution (NaClO, with 4% available chlorine), β-nicotinamide adenine dinucleotide (NADH), Tris-buffer HCl, methylene blue (MB), hydrogen peroxide (30%), ethanol (EtOH), methanol (MeOH), acetonitrile, formic acid, methylene blue (MB), sulfuric acid, formic acid, sodium chloride, tribasic sodium phosphate dodecahydrate, potassium chloride, sodium nitrite, sodium bicarbonate, monobasic potassium phosphate, and dibasic sodium phosphate were purchased from Sigma-Aldrich (St. Louis, MO, USA). L-tryptophan was obtained from Fisher Scientific (Pittsburgh, PA, USA). For all chromatographic analyses, samples and solvents were filtered using, respectively, 0.22 and 0.45 μm membranes, both from Millipore (Billerica, MA, USA). Ultrapure water was obtained from the Milli-Q system (Millipore Corp., Milford, MA, USA).

### 2.2. Arrabidaea chica Leaves

The leaves of *A. chica* (≈250 g), collected from five different plants belonging to the Active Germplasm Bank of *EMBRAPA Amazonia Oriental*, located in Belém, Pará, Brazil (01°26′14.7″ S and 48°26′52.2″ W), were freeze-dried (Liotop, L101, São Paulo, Brazil), ground in a knife mill, vacuum packed in plastic bags, and stored at −18 °C until analysis.

Access to the selected leaves was registered in the Brazilian National System for the Management of Genetic Heritage and Associated Traditional Knowledge (SisGen #A89EDD3).

### 2.3. Extracts of Arrabidaea chica Leaves

Three extracts from the leaves of *A. chica* were prepared, according to the methodology described by Chisté et al. [27] with some modifications, using the following green solvents: water, ethanol, and the ethanol/water mixture (1:1, *v*/*v*). The choice for these solvents considered the permissibility of residues in the extracts after evaporation, in accordance with Directive 95/45/EC of the Commission of European Communities [28].

The freeze-dried leaves of *A. chica* (3 g) were subjected to UAE with each green solvent in an ultrasonic bath (QUIMIS—model 03350, Diadema-SãoPaulo/Brazil) for 5 min at room temperature (25 °C), and fixed ultrasonic frequency at 25 KHz, at a solid/liquid ratio of 1:10 (*w*/*v*). After the UAE procedure, the extracts were centrifuged (Heraeus multifuge x 1R Thermo Electron Led GMBH, Göttingen, Germany) at 11,648× *g* for 5 min. The extraction procedure was repeated seven times for each solvent and the supernatants were combined after vacuum filtration. The extracts containing ethanol in the composition were subjected to evaporation at reduced pressure in a rotary evaporator (T < 38 °C). Water extract and the remaining water in the ethanol/water extract were frozen and freeze-dried. All the dried extracts were sealed under N_2_ flow and stored at −18 °C under light-free conditions, until analysis. The extractions were carried out in triplicate (*n* = 3).

### 2.4. HPLC-DAD Determination of Phenolic Compounds in the A. chica Extracts

The phenolic compound compositions of *A. Chica* extracts were determined by high-performance liquid chromatography (HPLC), coupled with a diode array detector (DAD) on an Agilent HPLC (model Agilent 1260 Infinity, Santa Clara, CA, USA) equipped with a quaternary pump (G1311C), an automatic injector (G7129), an oven (G1316A), and a DAD detector (G1328C).

The *A. chica* extracts were analyzed after solubilizing 10 mg of each dried extract in methanol/water (80:20, *v*/*v*) and the phenolic compounds were separated on a C_18_ Synergi Hydro column (Phenomenex, Torrance, CA, USA, 4 μm, 250 × 4.6 mm), at a temperature of 29 °C at 0.9 mL/min, with a linear gradient consisting of water–formic acid (99.5:0.5, *v*/*v*) and acetonitrile/formic acid (99.5:0.5, *v*/*v*) [29]. The UV–visible (UV–vis) spectra were recorded from 200 to 600 nm, and chromatograms were processed at 270, 320, and 360 nm. The phenolic compounds were identified by combining the following information: elution order, retention time in the C_18_ column, comparison with authentic standards analyzed under the same conditions, and UV–vis spectra compared to the phenolic compounds previously identified for *A. chica* leaves by LC-MS in our research group [20]. The phenolic compounds were quantified using six-point analytical curves (3 to 100 μg/mL in duplicate) of scutellarin (*r*^2^ = 0.99, limit of detection (LOD) = 0.14 μg/mL, and limit of quantification (LOQ) = 0.41 μg/mL), scutellarein (*r*^2^ = 0.98, LOD = 0.23 μg/mL, and LOQ = 0.69 μg/mL), and apigenin (*r*^2^ = 0.99, LOD = 0.17 μg/mL and LOQ = 0.51 μg/mL). The parameters of the analytical curves (standard deviation and the slope) were used to calculate the LOD and LOQ values [30]. The phenolic compound contents were expressed as mg/g of dried extracts (dry basis, d.b.), considering three independent extraction procedures (*n* = 3).

### 2.5. In Vitro Scavenging Capacity Determination against ROS and RNS

The ROS and RNS scavenging assays were carried out at 37 °C, in a microplate reader (Synergy HT, BioTek, Winooski, VT, USA) equipped with a thermostat using fluorescence, absorbance, or chemiluminescence modes of detection. Each antioxidant assay corresponds to, at least, four individual experiments, in triplicate, using five concentrations (0.03 to 500 µg/mL). The dried extracts of *A. chica* were dissolved in ethanol/water (1:1, *v*/*v*) for all the assays, and analyzed immediately to avoid the degradation of bioactive compounds. Quercetin (0.001 to 30 µg/mL) and scutellarein (0.10–500 µg/mL) were used as positive controls in the O_2_^•−^, HOCl, ^1^O_2_ and ONOO^−^ scavenging assays, while scutellarein and ascorbic acid (15 to 500 µg/mL) were used for H_2_O_2_. IC_50_ values (µg/mL) were calculated from curves of antioxidant concentrations versus the inhibition percentage using Origin Pro 8 software (OriginLab Corporation, Northampton, MA, USA). Before carrying out each assay, additional tests to check interference effects among the *A. chica* extracts and the solvents with the used probes or selected wavelengths were carried out and no interference was observed for the assay conditions.

#### 2.5.1. Superoxide Anion Radical (O_2_^•−^) Scavenging Assay

*A. chica* extracts were evaluated in relation to their capacity to scavenge O_2_^•−^, using a non-enzymatic system containing NADH/PMS/O_2_ [31]. This system is able to produce O_2_^•−^, which reduces NBT to a purple diformazan. The reaction mixtures in the microplate wells contained the following reagents at final concentrations (in a final volume of 300 µL): NADH (166 µM), NBT (43.3 µM), PMS (2.7 µM), the extracts dissolved in EtOH/H_2_O solution (1:1, *v*/*v*), and quercetin dissolved in DMSO. NADH, NBT, and PMS were dissolved in 19 mM phosphate buffer, pH 7.4. The inhibition percentage of NBT reduction to diformazan, by the extract/standards, was monitored by spectrophotometry at 560 nm after 10 min of plate introduction.

#### 2.5.2. Hydrogen Peroxide (H_2_O_2_) Scavenging Assay

The scavenging capacity of *A.chica* extracts against H_2_O_2_ was determined by monitoring the inhibition of chemiluminescence resulting from the H_2_O_2_-induced oxidation of lucigenin [31]. The reaction mixtures in the wells contained the following reagents at final concentrations (in a final volume of 250 µL): 50 mM Tris-HCl buffer (PH 7.4), lucigenin (0.8 mM) dissolved in Tris-HCl buffer, extracts of *A. chica,* and 1% (*w*/*w*) H_2_O_2_. The chemiluminescence signal was detected in the microplate reader after 5 min of plate introduction. Results were expressed as an inhibition percentage of the H_2_O_2_-induced oxidation of lucigenin.

#### 2.5.3. Hypochlorous Acid (HOCl) Scavenging Assay

The scavenging capacity of *A. chica* extracts against HOCl was determined by monitoring the inhibition of HOCl-induced oxidation of DHR to rhodamine 123 [31]. HOCl was prepared by adjusting a 1% (*v*/*v*) NaClO solution to pH 6.2 with H_2_SO_4_ 10% (*v*/*v*, followed by HOCl quantification by spectrophotometry at 235 nm, using the molar absorption coefficient of 100 M^−1^·cm^−1^ [32]. The reaction mixture was composed of the following reagents at the indicated final concentrations (final volume of 300 μL): DHR (5 μM), HOCl (5 μM), and *A. chica* extracts. The fluorescence signal, at an emission wavelength at 528 ± 20 nm and excitation at 485 ± 20 nm, was detected in the microplate reader immediately after plate insertion. Results were expressed as an inhibition percentage of the HOCl-induced oxidation of DHR.

#### 2.5.4. Peroxynitrite Anion (ONOO^−^) Scavenging Assay

The scavenging capacity of the *A. chica* extracts against ONOO^−^ was determined by monitoring the inhibition of the ONOO^−^-induced oxidation of DHR to fluorescent rhodamine 123 [32]. ONOO^−^ was synthesized as described by [32]. The reaction mixture was composed of the following reagents at the indicated final concentrations (final volume of 300 μL): DHR (5 μM), extract or standard, and ONOO^−^ (600 nM). The fluorescence signal, at an emission wavelength at 528 ± 20 nm and excitation at 485 ± 20 nm, was detected in the microplate reader after a 2 min incubation period. In a set of parallel experiments, assays were conducted in the presence of 25 mM NaHCO_3_ to simulate physiological conditions of CO_2_ concentration. The results were expressed as an inhibition percentage of the ONOO^−^-induced oxidation of DHR.

#### 2.5.5. Peroxyl Radical Scavenging Assay (ROO^•^) (ORAC)

ROO^•^ was generated by the thermodecomposition of AAPH at 37 °C, and the ROO^•^ scavenging capacity was measured by monitoring the effects of *A. chica* extract on the inhibition of fluorescence decay, due to fluorescein oxidation, induced by ROO^•^ [33,34]. The reaction mixtures consisted of the following reagents at the final concentrations (final volume of 200 μL): fluorescein (61.2 nM), AAPH solution (19.1 mM), and different concentrations of *A. chica* extracts dissolved in 75 mM phosphate buffer (pH 7.4). Trolox was used as a positive control. The fluorescence signal, at the emission wavelength of 528 nm with excitation at 485 nm, was monitored every minute until the total decay of fluorescence. The relative ability to capture ROO^•^ was expressed as the ratio between the slope of the curve of each extract or positive control and the slope obtained for trolox, as proposed by [34].

#### 2.5.6. Singlet Oxygen (^1^O_2_) Quenching Assay

The scavenging capacity of *A. chica* extracts and positive controls (quercetin and scutellarein) to inhibit ^1^O_2_ was evaluated according to the method described by De Siqueira et al. [20]. The ^1^O_2_ was generated, at room temperature (25 °C) and under atmospheric air, via the direct sensitization of methylene blue (MB) using a 75W incandescent lamp, used as an excitation source, and two filters (red and orange) were placed between the excitation source and the cuvette containing the reactants (*A. chica* extratcs, L-tryptophan, and MB), to excite MB only. The reaction was monitored by spectrophotometry, in the range of 200–800 nm, for 20 min, and the absorbance of tryptophan was recorded at 219 nm. The kinetic data obtained from the tryptophan absorbance decay were fitted to a first-order reaction to calculate the rate constants. The protection percentage of the *A. chica* extract, or the positive controls, against the oxidative damage of ^1^O_2_ was calculated through Equation (1).

(1) $$Protection\;\left(\%\right)=\frac{K_{obs}^{TRP}-K_{obs}^{TRP+antioxidant}}{K_{obs}^{TRP}}\times 100\;$$

where $K_{obs}^{TRP}\;$is the rate constant for the observed pseudo-first-order reaction fitted to the TRP decay curve (obtained in the blank experiment) and $K_{obs}^{TRP+antioxidant}$ is the rate constant for the observed pseudo-first-order reaction fitted to the decay curve of TRP in the presence of the antioxidant compound.

### 2.6. Statistical Analysis

The IC_50_ values (mean ± standard deviation) were subjected to ANOVA analysis of variance and the means were classified by Tukey’s test at the 95% significance level using Statistica 7.0 software (Statsoft Inc., Tulsa, OK, USA). Analytical curves were plotted by linear regression (*p* < 0.05) using Origin 8 Software (OriginLab Corporation, Northampton, MA, USA).

## 3. Results and Discussion

### 3.1. Phenolic Compounds Composition of Arrabidaea chica Extracts

Any modification in the composition of a selected solvent alters its polarity, and consequently promotes changes in the phenolic compounds’ composition during extraction procedures [31]. Considering the low toxicity, good extraction yield, safety for human consumption, and its application in the food industry, ethanol, ethanol/water mixture (1:1, *v*/*v*), and water were used as green solvents. Thus, in addition to the cavitation promoted by the UAE procedure that facilitates bioactive compound extraction from plant tissues, the composition of phenolic compounds identified in the extracts of *A. chica* leaves was the result of their solubility in each solvent (Table 1).

**Figure 1:**
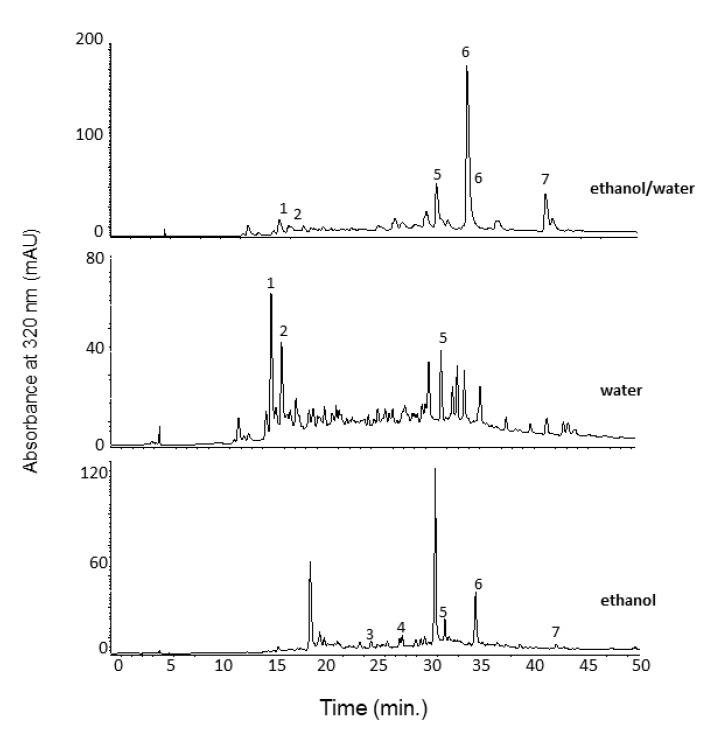
HPLC-DAD chromatogram at 320 nm of phenolic compounds of the extracts of *Arrabidaea chica* leaves. Peak characterization is given in Table 1.

HPLC analysis of the freeze-dried extracts of *A. chica* leaves allowed the separation and quantification of seven phenolic compounds (Figure 1). However, variations were observed among the extracts regarding the individual compounds. The highest levels of phenolic compounds were found in the ethanol/water (1:1, *v*/*v*) extract (Table 1), about three times higher than the extracts obtained with ethanol and water separately.

The mixture of solvents of different polarities, such as ethanol (which has medium polarity) and water (which is a strong polar solvent), promotes high efficiency in the extraction of a range of phenolic compounds of different degrees of polarity [35,36,37].

In addition, mixing water with organic solvents was reported to increase the efficiency of phenolic compound extraction from dry samples, since it allows the hydration of dry particles and the swelling of plant tissues, thus favoring the penetration of the organic solvent into the plant matrix [38] and consequently increasing mass transfer by molecular diffusion [39]. Furthermore, ethanol breaks the bonds between the sample matrix and phenolic compounds, which increases the recovery of these compounds [39].

Unlike the extract of *A. chica* leaves using methanol/water (8:2, *v*/*v*), as previously published by our research group [20], which showed scutellarin as the major compound, in the ethanol/water extract (1:1, *v*/*v*) (Figure 1), only its aglycone form (scutellarein) was identified, comprising about 57% of the total area of the phenolic compounds (Table 1). Both the compounds were found in the extract obtained with ethanol, with scutellarein found at the highest concentration (41%).

On the other hand, in the extract obtained with water, the flavonoid concentrations were lower (glucuronyl flavone derivative) or not detected (scutellarin, scutellarein, and apigenin). However, water extract presented the highest levels of phenolic acids (isomers of ferulic acid glycosides), confirming the selectivity of water for compounds of high polarity in the composition of *A. chica* leaves.

The ethanol–water extract of *A. chica* leaves showed that the total phenolic compound contents were ≈10 times lower than that found in the hydromethanolic extract of the same plant (21.5 mg/g) [20]. By comparing extracts obtained from other vegetables, it showed lower values than the hydroalcoholic extract of artichoke leaves (*Cynara cardunculus*) (73 mg/g), similar to *Eryngium foetidum* (9.99 mg/g) [40] and higher than extracts obtained from the peel (5.4 mg/g) and pulp (1.8 mg/g) of *Antrocaryon amazonicum* fruits [41].

### 3.2. ROS- and RNS-Scavenging Capacity of the Arrabidaea chica Extracts

**Figure 2:**
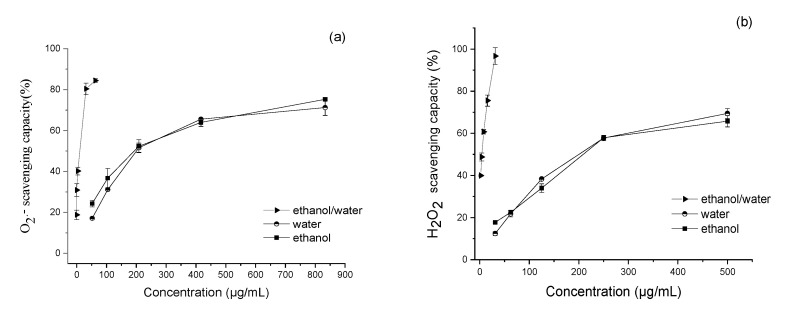

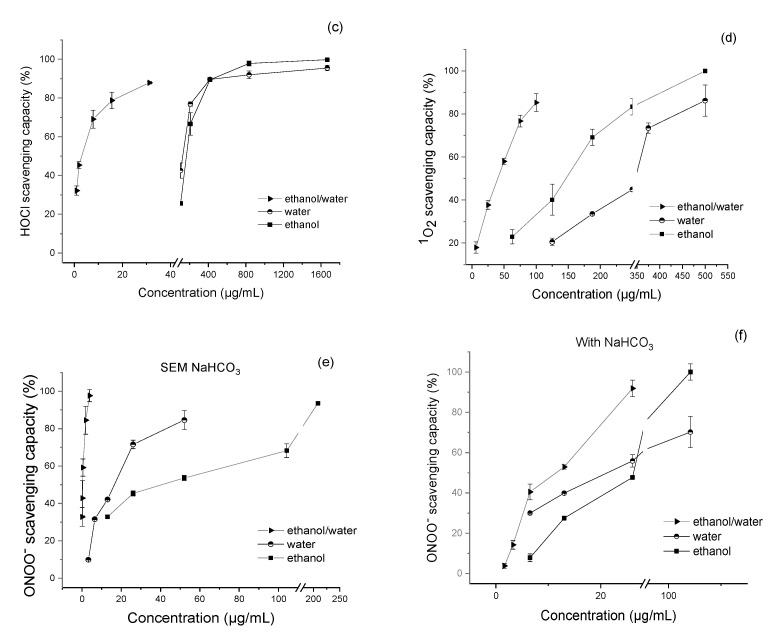
Concentration-dependent behavior of extracts of *Arrabidaea chica* leaves against (**a**) superoxide anion radicals (O_2_^•−^), (**b**) hydrogen peroxide (H_2_O_2_), (**c**) hypochlorous acid (HOCl), (**d**) singlet oxygen (^1^O_2_), and (**e**) peroxynitrite anion (ONOO^−^) in the absence and (**f**) presence of NaHCO_3_. Each point represents the values obtained by four experiments, at five concentrations, carried out in triplicate (mean ± standard deviation).

*A. chica* extracts and scutellarein (authentic standard) could scavenge all the ROS and RNS tested, with IC_50_ values at low µg/mL ranges (Table 2) and in a concentration-dependent manner (Figure 2).

In general, the extract obtained with ethanol/water (1:1, *v*/*v*), which presented the highest contents of phenolic compounds, exhibited higher antioxidant efficiency against all the ROS and RNS tested, with lower IC_50_ values (Table 2). Interestingly, all the extracts showed the same scavenging capacity against ROO^•^, with five times higher values than trolox (positive control).

In relation to O_2_^•−^, the extract of *A. chica* obtained with ethanol/water (1:1, *v*/*v*) was significantly more effective than those obtained with ethanol and water, and it showed higher scavenging capacity than quercetin (positive control) (Table 2), as well as other medicinal plants, such as *Castanea sativa* (13.60 µg/mL) and *Quercus robur* (11.00 µg/mL) [42], and was about five times more efficient than the leaves of *Vismia cauliflora* (medicinal plant from the Amazon biome) (54.00 µg/mL) [12]. The high efficiency of the ethanol/water extract (1:1, *v*/*v*) may be associated, in addition to the presence of scutellarein (major compound), to the synergy with other phenolic compounds, given the fact that this extract was considered a more effective scavenger of O_2_^•−^ when compared with scutellarein (about 10 times).

O_2_^•−^ can be formed enzymatically and chemically from triplet oxygen (molecular oxygen, ^3^O_2_). This ROS is very important in the reduction of oxygen to generate other reactive species, such as H_2_O_2_, hydroxyl radicals (HO^•^), and ^1^O_2_. In food systems, it can be generated, in addition to the action of enzymes, such as xanthine oxidase, by ohmic food processing, gamma irradiation, microwaves, and pulsed electric field, and via the reaction of ^3^O_2_ with the decomposition products of some azo compounds, such as azo dyes [7,43].

In most organisms, O_2_^•−^ is converted into H_2_O_2_ with the enzyme superoxide dismutase (SOD). Although H_2_O_2_ is not a free radical, it has a reactive potential, and in the presence of metal ions, it produces HO^•^, which is an oxidizing species with very high reactivity [44,45]. Likewise, in foods, H_2_O_2_ can indirectly impart the loss of quality, since HO^•^ can act as an initiator of lipid peroxidation [7]. The ethanol/water extract (1:1, *v*/*v*) of *A. chica* was the most efficient in scavenging H_2_O_2_ among the tested extracts, with higher efficiency than scutellarein and ascorbic acid, both used as positive controls (Table 2). On the other hand, all the tested extracts also showed high efficiency when compared to other plant extracts, with IC_50_ values about 1 to 66 times lower than those of *Vismia cauliflora* leaves (289.00 µg/mL) [12] and 2 to 88 times of extracts of *Juglans regia* (383 µg/mL) [42].

Another important ROS, HOCl, can be generated in the presence of H_2_O_2_, produced by activated neutrophils and monocytes, where the enzyme myeloperoxidase (MPO) catalyzes the oxidation of chloride ion (Cl^−^). HOCl is highly harmful and causes oxidation and chlorination reactions in biological systems [46]. It is considered a potent pro-inflammatory agent and consequently associated with a number of diseases resulting from chronic and degenerative inflammation and various types of cancer [47,48]. In this study, the ethanol/water extract (1:1, *v*/*v*) was also the most potent HOCl scavenger, followed by the water and ethanol extracts, which is even more efficient than the quercetin and scutellarein (Table 2). Likewise, it showed higher scavenging efficiency than other hydrophilic extracts of Amazonian fruits, such as *Byrsonima crassifolia* (10 μg/mL) [49] and *Solanum sessiliflorum* (13 μg/mL) [50], and the IC_50_ value was close to the values reported for extracts of artichoke (*Cynara cardunculus*) leaves (3.7 to 4.7 µg/mL) [51].

Regarding the ability to quench ^1^O_2_, a highly reactive ROS that is frequently generated in both physiological and food systems, the ethanol/water extract (1:1, *v*/*v*) of *A. chica*, also showed the highest antioxidant capacity, yet was less effective than quercetin (1.88 µg/mL) and scutellarein (7.84 µg/mL). The ethanol–water extract presented an IC_50_ value close to the values reported for extracts of *Vismia cauliflora* leaves (27.00 µg/mL) [12] and *Cynara cardunculus* (29.00 µg/mL) [51], and was about seven times more efficient than *Solanum diploconos* (269 µg/mL), a native Brazilian fruit [4]. Natural extracts that exhibit high ^1^O_2_-quenching ability are highly desirable, since this ROS, induced by light and in the presence of photosensitizers such as chlorophyll, phaeophytins, riboflavin, myoglobin and heavy metals, may react with unsaturated fatty acids via non-radical pathways, as in photo-oxidation processes, to speed up lipid peroxidation [7,9]. As an example, the incidence of light on foods, such as beer, milk, and cheese, in the presence of riboflavin and other photosensitizers, can promote the formation of off-flavors due to the degradation of lipid and protein molecules [52]. Likewise, in biological organisms, photo-oxidation also contributes to oxidative damage, such as in skin diseases such as photoaging and photocarcinogenesis [9]. Thus, the incorporation of extracts with high scavenging capacity against ROS/RNS in food systems or even in cosmetic formulations is frequently seen as an effective strategy to delay direct oxidative reactions.

As another very important ROS highly associated with oxidative damages in both food and biological systems, ROO^•^ are originated in the propagation step during the chain reactions of lipid peroxidation. The presence of double bonds in unsaturated and polyunsaturated fatty acids of edible oils and other animal derived products makes them highly susceptible to oxidative damage, leading to the degradation of essential fatty acids and fat-soluble vitamins, promoting sensory changes and formation of chemical compounds with harmful effects on health, which compromises the nutritional quality and safety of foods [9,53]. Lipid autoxidation is observed in the presence of molecular oxygen and reactive species, and it involves a chain reaction that includes initiation, propagation, and termination steps. After initiation, where lipid radicals are formed from lipid molecules, propagation reactions take place, where the lipid-free radicals quickly react with molecular oxygen-generating ROO^•^, which produce hydroperoxides (ROOH) after abstracting hydrogen from another intact molecule of unsaturated fatty acid [54]. Furthermore, in the presence of transition metals, such as Fe and Cu, or in light or high temperatures, ROOH can be decomposed into alkoxyl radicals (RO^•^), and then form by-products of lipid oxidation, such as ketones, aldehydes, acids, esters, and other derived compounds (such as malonaldehyde) [7].

A common practice in the oilseed industries is the addition of synthetic antioxidant compounds, such as butylhydroxytoluene (BHT), butylhydroxyanisole (BHA), *tert*-butylhydroquinone (TBHQ), and propyl galatte (PG), to delay or inhibit the oxidative processes of edible oils [55,56]. However, there is an increasing demand in the food industry to incorporate antioxidants of natural origin in the processing of food products, as replacements to the artificial ones, to accomplish the increased world tendency for the development of healthier products as part of healthy habits. In our study, all the *A. chica* extracts presented high potential to be used as a natural alternative to increase the stability of food systems. All the tested extracts exhibited the same scavenging capacity against ROO^•^ (Table 2), and they were 5 times more efficient than trolox (positive control) and about 33 times more efficient than scutellarein, which can raise the hypothesis of synergy between the other phenolic compounds in the extracts. When compared to other plant species, the *A. chica* extracts were considered effective scavengers of ROO^•^, about 26 times more effective than the extracts of pulp + skin (0.19, S_sample_/S_trolox_) and seeds (0.16, S_sample_/S_trolox_) of *Citharexylum solanaceum* [57], and were 16 times more efficient than the hydroalcoholic extract of artichoke leaves (*Cynara cardunculus*) (0.31, S_sample_/S_trolox_) [51].

Regarding RNS, ONOO^−^ is a strong oxidizing and nitrosative agent, which can affect the quality of foods, with O_2_^•−^ and nitric oxide (^•^NO) being precursors. ONOO^−^ can initiate lipid oxidation in foods, with the formation of free radicals that can lead to the loss of essential fatty acids, vitamins, and proteins [58,59]. In biological systems, ONOO^−^ can also cause nitrosative damage to biomolecules, including DNA, proteins and lipids, and enzymatic inactivation, among other reactions [45,52]. In the scavenging capacity assays of ONOO^−^, it is also important to determine the ability to scavenge ONOO^−^ in the presence of NaHCO_3_ as a function of the predominance of the reaction between ONOO^−^ and CO_2_ under physiological conditions [60].

Regarding the scavenging capacity of *A. chica* extracts against ONOO^−^, the ethanol–water extract also showed higher efficiency than the other extracts, in the presence and absence of NaHCO_3_, followed by the water extract (Table 2). The ethanol–water extract was less efficient than quercetin (positive control), but was more efficient than other plant extracts, such as the extracts from the whole fruit of *Solanum diploconos* [4], presenting IC_50_ about eighty times lower values in the absence of NaHCO_3_ (27.8 µg/mL) and twice in the presence of NaHCO_3_ (27.3 µg/mL). Additionally, the scavenging capacity of the ethanol–water extract was about seventeen times higher than that of the extracts of *Vismia cauliflora* leaves (5.8 µg/mL) in the absence of NaHCO_3_ [12].

In this study, the *A. chica* extracts showed high levels of phenolic compounds, and one of the advantages of using plant extracts as natural antioxidants might be related to the synergistic effects between the constituents of the extracts. The fact that the ethanol/water extract (1:1, *v*/*v*) of *A. chica* was as effective as the positive controls against ROS and RNS highlights the interest in its use as alternative components in food formulations. In addition, another advantage that lies with the use of natural antioxidants is the replacement of synthetic ones, which express safety concerns due to their suspected carcinogenic effects [61].

## 4. Conclusions

This is the first report concerning the ability of *A. chica* leaf extracts to scavenge physiologically relevant ROS and RNS, namely HOCl, H_2_O_2_, O_2_^•−^, and ONOO^−^. All the *A. chica* extracts and scutellarein were able to scavenge all the reactive species tested in a concentration-dependent manner. Among the extracts, the one produced with ethanol–water as green solvent presented the highest content of phenolic compounds, where scutellarein was the major compound, and exhibited the highest scavenging capacity against all the ROS and RNS tested.

The antioxidant potential herein investigated may be useful to delay or minimize the oxidative damages induced by the overproduction of ROS/RNS in both physiological and food systems.

## Figures and Tables

**Table 1:** Profile of phenolic compounds from extracts of *A. chica* determined by HPLC-DAD.

Peak	Phenolic Compound *	t_R_ (min) ^a^	λ_max_ (nm) ^b^	Concentration (mg/g Extract) ^c^		
				EtOH/H_2_O	H_2_O	EtOH
1	Feruloyl hexose (isomer 1) ^d^	15–16.3	313	0.85 ± 0.03	1.71 ± 0.06	nd
2	Feruloyl hexose (isomer 2) ^d^	16.6–17.5	309	0.49 ± 0.05	1.28 ± 0.03	nd
3	Feruloyl derivative ^d^	24.7	273, 327	nd	nd	0.45 ± 0.01
4	Scutellarin ^e^	27.7	282, 334	nd	nd	0.67 ± 0.09
5	Flavone glucuronyl derivative ^d^	31.6–32.6	275, 328	2.18 ± 0.36	0.82 ± 0.05	0.88 ± 0.07
6	Scutellarein ^d^	34.6–35.5	282, 337	6.79 ± 0.59	nd	1.51 ± 0.13
7	Apigenin ^f^	42.3–43.3	267,293, 337	1.49 ± 0.09	nd	0.09 ± 0.02
	**Total sum (mg/g)**			**11.80 ± 1.13**	**3.81 ± 0.15**	**3.62 ± 0.25**

* Tentative identification based on the retention time on C_18_ column, UV–visible spectra and comparison with the identification previously carried out by our research group for *A. chica* leaves (De Siqueira et al. 2019 [20]). ^a^ Retention time (t_R_) on C_18_ column. ^b^ Solvent: gradient of water with 0.5% formic acid and acetonitrile with 0.5% formic acid. ^c^ Mean ± standard deviation (*n* = 3, dry basis). The peaks were quantified as equivalent of ^d^ scutellarein, ^e^ scutellarin, and ^f^ apigenin. Abbreviations: nd, not detected; EtOH/H_2_O, ethanol/water (1:1, *v*/*v*); H_2_O, water; EtOH, ethanol.

**Table 2:** Scavenging capacity of extracts of *A. chica* leaves and standard compounds against reactive oxygen species (ROS) and reactive nitrogen species (RNS).

Extract/ Compound	ROS					RNS	
	IC_50_ (μg·mL^−1^) *				S_sample_/S_trolox_	IC_50_ (μg·mL^−1^)	
	O_2_^•−^	H_2_O_2_	HOCl	^1^O_2_	ROO^•^	ONOO^−^	
						Absence of NaHCO_3_	Presence of NaHCO_3_
H_2_O	204 ± 13 ^a^	198 ± 4 ^a^	127 ± 9 ^b^	271 ± 1 ^a^	5.00 ± <0.01 ^a^	16.5 ± 0.2 ^b^	21 ± 1 ^b^
EtOH/H_2_O	10 ± 1 ^c^	4.3 ± 0.5 ^d^	2.9 ± 0.3 ^c^	35 ± 6 ^c^	5.00 ± <0.01 ^a^	0.34 ± 0.07 ^d^	11.1 ± 0.7 ^c^
EtOH	196 ± 2 ^a^	210 ± 9 ^a^	166 ± 14 ^a^	143 ± 8 ^b^	5.00 ± <0.01 ^a^	40.3 ± 0.6^a^	28.3 ± 0.9 ^a^
**Positive control**							
Scutellarein	107 ± 9 ^b^	151 ± 5 ^b^	4.40 ± 0.10 ^c^	7.8 ± 0.2 ^d^	0.15 ± <0.01^b^	7 ± 0.4^c^	4.7 ± 0.4 ^d^
Quercetin	12.9 ± 0.5 ^c^	-	13 ± 1 ^c^	1.9 ± 0.1 ^d^	-	0.01± < 0.01^d^	0.01 ± <0.01 ^e^
Ascorbic acid	-	41 ± 7 ^c^	-	-	-	-	-
Trolox					1.00		

* IC_50_, inhibitory concentration in vitro to decrease the oxidizing effect of each reactive species by 50% (mean ± standard deviation) (*n* = 3, dry basis). Sample = slope for the curve of *A. chica*. S_Trolox_ = slope for the trolox curve. H_2_O = water; EtOH/H_2_O = ethanol–water; EtOH = ethanol. Means at the same column with the same lowercase superscript letters are statistically equal at 95% significance (Tukey’s test).

## Data Availability

The data presented in this study are available in the article.
